# Dynamics of Health Agency Response and Public Engagement in Public Health Emergency: A Case Study of CDC Tweeting Patterns During the 2016 Zika Epidemic

**DOI:** 10.2196/10827

**Published:** 2018-11-22

**Authors:** Shi Chen, Qian Xu, John Buchenberger, Arunkumar Bagavathi, Gabriel Fair, Samira Shaikh, Siddharth Krishnan

**Affiliations:** 1 Department of Public Health Sciences University of North Carolina at Charlotte Charlotte, NC United States; 2 School of Communications Elon University Elon, NC United States; 3 Department of Computer Sciences University of North Carolina at Charlotte Charlotte, NC United States

**Keywords:** Centers for Disease Control and Prevention, public engagement, Twitter, time series analysis, Zika epidemic, social media, twitter, infodemiology, infoveillance

## Abstract

**Background:**

Social media have been increasingly adopted by health agencies to disseminate information, interact with the public, and understand public opinion. Among them, the Centers for Disease Control and Prevention (CDC) is one of the first US government health agencies to adopt social media during health emergencies and crisis. It had been active on Twitter during the 2016 Zika epidemic that caused 5168 domestic noncongenital cases in the United States.

**Objective:**

The aim of this study was to quantify the temporal variabilities in CDC’s tweeting activities throughout the Zika epidemic, public engagement defined as retweeting and replying, and Zika case counts. It then compares the patterns of these 3 datasets to identify possible discrepancy among domestic Zika case counts, CDC’s response on Twitter, and public engagement in this topic.

**Methods:**

All of the CDC-initiated tweets published in 2016 with corresponding retweets and replies were collected from 67 CDC–associated Twitter accounts. Both univariate and multivariate time series analyses were performed in each quarter of 2016 for domestic Zika case counts, CDC tweeting activities, and public engagement in the CDC-initiated tweets.

**Results:**

CDC sent out >84.0% (5130/6104) of its Zika tweets in the first quarter of 2016 when Zika case counts were low in the 50 US states and territories (only 560/5168, 10.8% cases and 662/38,885, 1.70% cases, respectively). While Zika case counts increased dramatically in the second and third quarters, CDC efforts on Twitter substantially decreased. The time series of public engagement in the CDC-initiated tweets generally differed among quarters and from that of original CDC tweets based on autoregressive integrated moving average model results. Both original CDC tweets and public engagement had the highest mutual information with Zika case counts in the second quarter. Furthermore, public engagement in the original CDC tweets was substantially correlated with and preceded actual Zika case counts.

**Conclusions:**

Considerable discrepancies existed among CDC’s original tweets regarding Zika, public engagement in these tweets, and actual Zika epidemic. The patterns of these discrepancies also varied between different quarters in 2016. CDC was much more active in the early warning of Zika, especially in the first quarter of 2016. Public engagement in CDC’s original tweets served as a more prominent predictor of actual Zika epidemic than the number of CDC’s original tweets later in the year.

## Introduction

The World Health Organization (WHO) has stated that health is one of the most fundamental human rights [1]. Social media have increasingly become critical venues for the public to seek, share, and discuss information about health and diseases. Owing to their low cost, easy access, and broad reach, social media have also been increasingly adopted by health professionals and agencies to enhance public health communication [2]. For example, social media have been utilized to monitor food safety and food-borne pathogen outbreak, such as *Escherichia coli* O157 [3,4], to develop Web-based campaigns to quit smoking in different countries and regions (United States, Canada, and Hong Kong) with various social media platforms (Facebook, Twitter, and WhatsApp [5]); promote exercise, fitness, and healthy lifestyle (WeChat health campaign in China [6]; fitness campaign in New Orleans, LA [7]); raise public awareness and engagement regarding air quality and pollution [8]; and understand and monitor public discussion of controversial topics such as antimicrobial resistance [9].

Many government agencies and health officials (eg, WHO and US Centers for Disease Control and Prevention, CDC, as well as other local health departments) have also been adopting and utilizing social media to disseminate information, communicate with the public, and understand public opinions and concerns, especially during health emergency and crisis. Europe has developed a Web-based media and crisis communication framework for influenza [10]. The WHO and CDC utilized Twitter and Instagram during the Zika outbreak [11]. New York City monitored Zika, Hepatitis A, and Ebola discussion in social media and conducted risk communication with the general public [12].

Evidently, for many infectious disease epidemics, it has been demonstrated that Web-based discussion in social media can be an imperative indicator of the actual disease severity and help health officials to more accurately evaluate the time-sensitive epidemic situation when actual case counts are still being gathered and verified [13-15]. Time series analysis is a versatile and powerful modeling framework to link Web-based discussion and reveal the disease dynamics, as demonstrated by the extant research on various epidemics [16-18].

The 2016 Zika epidemic provides a great opportunity to investigate and evaluate the CDC’s role and responsiveness on social media. Zika was a relatively new infectious disease, which affected men and women, fetuses, and infants with multiple transmission routes. However, the general public usually had very little knowledge and understanding about it. In 2016, Zika caused 5168 confirmed noncongenital cases in the 50 states and Washington DC in the United States, and much higher case number in US territories [19]. Twitter is the major social media outlet for the CDC, with a total of 67 official CDC–associated Twitter accounts covering a wide variety of health- and disease-related topics. Former CDC director Dr. Tom Frieden was active on Twitter and hosted live Twitter chats with general public [20], including a recent 1-hour live chat regarding Zika in February 2016.

Despite CDC’s prominent Web-based presence and efforts, inaccurate information regarding Zika proliferated on social media and outperformed the CDC (and other legitimate sources such as the WHO) by a large margin [21]. Studies have shown a substantial topic discrepancy between public concern and the CDC’s response to Zika on Twitter [22-25]. Another less addressed aspect is the low rate of public engagement (measured by the number of retweets and replies) on social media, where social media should be a Web-based platform for public engagement and interaction [26], not just one-directional news outlet [8,27,28]. Furthermore, currently there is no study on the temporal variability in the CDC’s response to different epidemic stages of Zika for the entire year of 2016, its potential impact on public engagement, and quantification of information dissemination, as the CDC did not finalize and publish the complete 2016 Zika case counts in the entire United States until March 2018 [19].

Thus, there is a substantial knowledge gap in quantifying and understanding the interaction among Zika epidemic, the CDC’s dynamic response on social media (Twitter), and public engagement to the CDC’s effort, as well as potential discrepancy among these hierarchies during different stages of the Zika epidemic. More specifically, original CDC-initiated tweets regarding Zika represent the government agency’s responsiveness to the Zika epidemic. Retweets and replies to CDC’s original tweets quantify public engagement in the discourse about Zika in Twitter. Between the 2, retweets enhance Zika-related news and information discourse by replying information to other users, whereas replies imply more in-depth cognitive processing of this topic and contribute to the direct interaction with CDC [29].

To address these issues, this study aims to quantify the CDC’s responsiveness on Twitter and corresponding public engagement during different stages of the 2016 Zika epidemic. We then identify potential discrepancy among them using time series analysis and information theory measurements. The results and insights gained from this study will reveal the effectiveness of CDC’s efforts in disseminating information on social media and help develop more effective Web-based communication strategies to inform public and combat fake information in health-related topics.

## Methods

### Data Collection and Preparation

We collected all English tweets with the keyword “Zika” published between January 1, 2016 and December 31, 2016, using the Gnip Twitter application program interface. Corresponding retweets and replies received by these tweets were also collected. In addition, all tweets from 67 accounts affiliated with CDC in 2016 were collected. Zika case counts in the 50 US states and territories during the entire 2016 have been retrieved from the official CDC Zika case report website [29] and CDC’s final report of the 2016 Zika epidemic in the United States [19].

Four time series were extracted from the original tweets (both Zika-related and all tweets initiated by CDC), retweets, and replies (only to Zika-related CDC-initiated tweets). In addition, 2 additional time series of US Zika case counts (both 50 states and 50 states plus territories) were obtained [19]. Given that the dates of tweets, retweets, replies, and case counts were not entirely consistent (eg, the CDC may not tweet about Zika every day and may not publish case count on a regular basis), these time series were first standardized into weekly basis. The data were aggregated in weekly periods to ensure that each time series has the same 52 data points for further analysis and comparison. Monthly resolution was not adequate to perform successive time series analyses (because each quarter only had 3 data points) while daily resolution required an extra step of data interpolation (because each day did not necessarily had Zika tweets and case reports), and weekly basis was well balanced and should provide the highest signal-to-noise ratio in this study. To establish a baseline scenario, we computed the weekly number of tweets with any topic from all CDC accounts and identified the top topics tweeted by CDC in 2016. Using these data, we could calculate the ratio between weekly tweets with the keyword of Zika and all tweets from the CDC, which demonstrated the relative importance of Zika on the CDC’s social media agenda. This estimate also helped reveal and assess the CDC’s responsiveness to Zika at different stages of the epidemic.

### Univariate Time Series Analysis

Original Zika tweets from the CDC, corresponding retweets and replies, and Zika case time series were plotted, visualized, and examined for stationarity. After the initial screening, we discovered a substantial temporal variability in the number of original tweets, retweets, and replies, as well as Zika cases. None of these time series was stationary. To characterize such large temporal heterogeneity, we divided the entire year of 2016 into 4 quarters and performed further analysis within each quarter. Furthermore, we calculated the ratio between Zika tweets and all tweets from the CDC as a measurement to quantify the relative importance of Zika among various health-related topics from the CDC’s perspective.

These quarterly time series were first modeled as autoregressive integrated moving average (ARIMA) models to reveal any potential temporal characteristics such as linear trend, seasonality, or temporal autocorrelation [16]. The following equation:

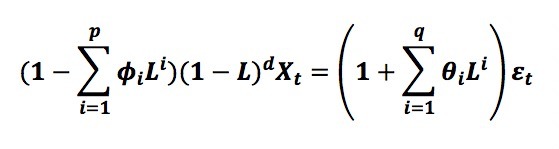



shows the form of an ARIMA model with variable *X*_*t*_, difference term *L*, and parameters (*p, d, q*) (Equation 1). The 3 parameters *p*, *d*, and *q* corresponded to autoregressive, differencing/integrated (L), and moving average components of the ARIMA model, respectively. The optimal model was then chosen by minimizing the Akaike Information Criteria (AIC) value among all possible competing models with different parameters. The Zika case counts were excluded from this analysis because most of the domestic Zika cases in 2016 were travel-related and could not be well characterized by the ARIMA model, and modeling the temporal dynamics of Zika was not an aim of this study.

### Multivariate Time Series Analysis

We calculated the lagged correlation between 2 time series using the cross-correlation function (CCF) at different stages represented by 4 quarters in 2016 to identify and quantify the potential temporal discrepancy among Zika case counts, CDC’s original tweets, and public engagement in these tweets (ie, retweets and replies to CDC’s tweets). Specifically, we compared time series of Zika case counts with that of original CDC tweets to understand the CDC’s responsiveness to the disease outbreak. In addition, time series of Zika case counts and that of retweets and replies were compared with discovered different levels of public engagement in reaction to the Zika epidemic. Their respective CCFs were computed for each of the 4 quarters in 2016. Given that the original CDC tweets were always highly correlated with retweets and replies, we also evaluated the dynamic change of public engagement by calculating the ratio between the number of CDC’s original Zika tweets and the number of retweets or replies across different stages. In addition, we calculated the mutual information between 2 time series using Dirichlet-multinomial pseudo count Bayesian estimate of Shannon entropy, a more informative metric than the CCF to reveal the potential mutual information between 2 time series and quantify whether the number of original CDC tweets about Zika and retweets and replies received by them had adequate mutual information with actual Zika case counts.

We constructed the ARIMA with External Variable (ARIMAX) model for original CDC tweets, retweets, and replies in each quarter of 2016, respectively. The ARIMAX model was a multivariate extension of the ARIMA model and incorporated an effective external variable (ie, *Y*_*t*_ , representing a time series of Zika case counts in this study):

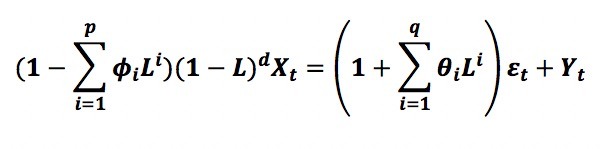



The univariate ARIMA model and multivariate ARIMAX model were then compared to see whether including external variable actually increased the model performance by decreasing the AIC value. The ARIMAX model was constructed on the basis of the corresponding optimal ARIMA model in the univariate time series analysis section. In other words, ARIMAX and ARIMA models should have exactly the same *p, d,* and *q* parameter values to correctly assess the effect of the external variable. This revealed whether public engagement in CDC’s original tweets significantly corresponded to the domestic Zika epidemic. We then tested whether the number of original CDC tweets, retweets, or replies could serve as an imperative indicator of actual Zika case (or *vice versa*) in different stages by applying the Granger causality test. The terms that needed to be first differenced in the Granger test were determined from the corresponding ARIMA or ARIMAX model (ie, where parameter *d* is nonzero).

## Results

### Descriptive and Univariate Time Series Analysis Results

**Figure 1:**
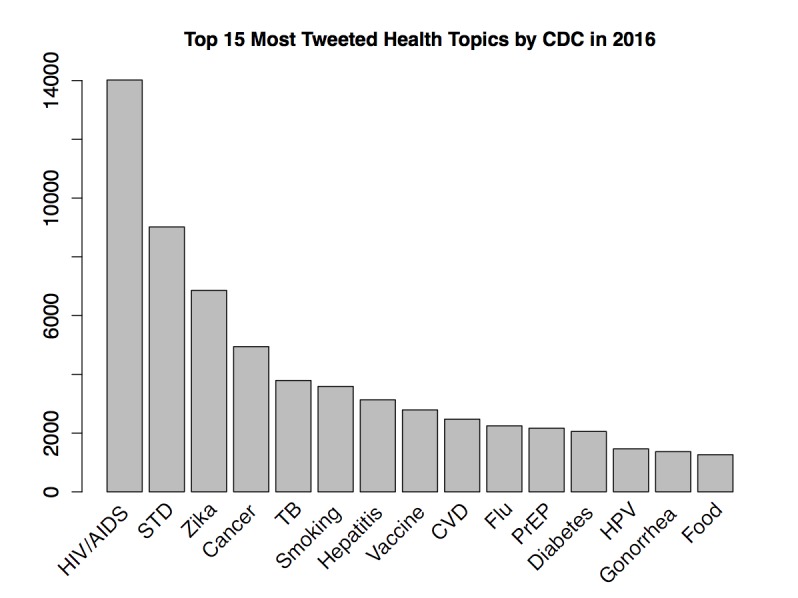
The top 15 most tweeted health topics by the Centers for Disease Control and Prevention (CDC) in 2016. STD: sexually transmitted disease TB: tuberculosis; CVD: cardiovascular disease; PreP: Pre-exposure prophylaxis; HPV: Human papillomavirus.

**Figure 2:**
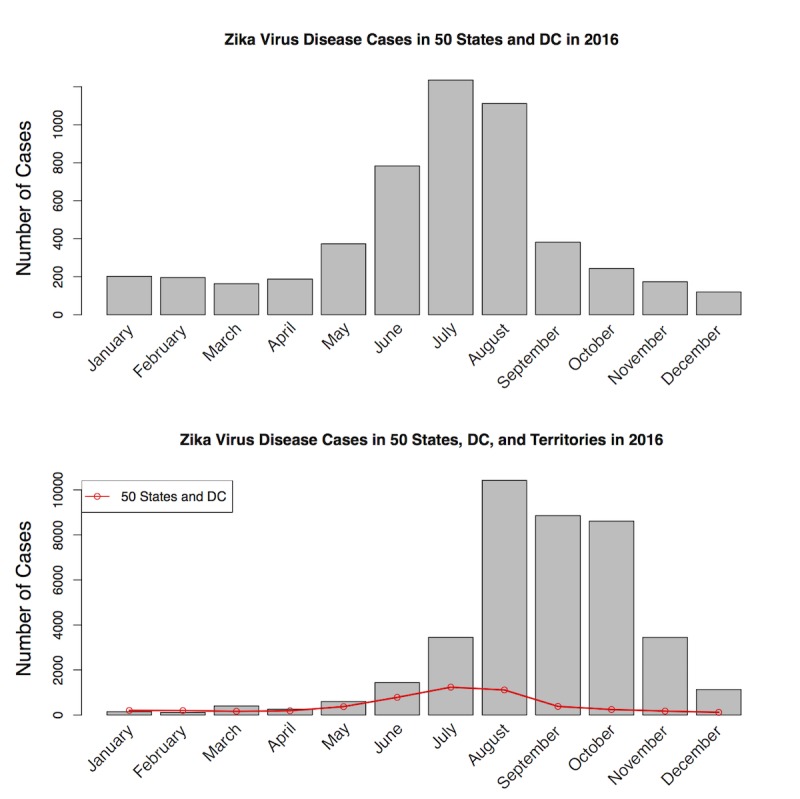
The time series of Zika tweets from the Centers for Disease Control and Prevention (CDC), corresponding retweets, replies, and all original tweets from the CDC in 2016.

Among all tweets sent by the CDC in 2016, Zika was the third most tweeted health topic, totaling >6000 tweets (including 4000 original tweets and another 2000 retweets by other CDC–associated Twitter accounts), and was just behind HIV/AIDS and sexually transmitted disease in entire 2016 (Figure 1). As there might be overlap between topics (eg, Zika/sexually transmitted disease, Zika/Vaccine, HIV AIDS/Pre-exposure prophylaxis, HPV/Vaccine, etc), a specific tweet could belong to multiple topics. Thus, Zika was a highly ranked and important health topic in 2016 according to the CDC. Among all 67 CDC–associated Twitter accounts, 21 tweeted about Zika in 2016. More than 60% (3663/ 6104) of Zika-related tweets were posted by @CDCgov, @CDCTravel, @CDCGlobal, and @CDCEmergency; these 4 were also the most active Twitter accounts that disseminated Zika-related information consistently through all 4 quarters in 2016. Although Zika was one of the hot topics tweeted by the CDC, there was substantial temporal heterogeneity in the CDC’s tweeting pattern regarding Zika. More than 84.0% (5130/6104) of all Zika tweets were published in the first quarter of 2016, with 5.6% (342/6104), 7.5% (458/6104), and 2.4% (146/6104) for the subsequent quarters, respectively (Figure 2). The top left of Figure 2 shows the number of all tweets sent from all CDC–associated Twitter accounts during 2016 (solid black line) and Zika-related tweets (dashed blue line); the top right shows the number of Zika-related tweets (solid black line) and Zika case counts in 50 states and DC (solid red line); the bottom left shows retweets to CDC’s Zika tweets; and the bottom right shows replies to CDC’s Zika tweets. As a comparison of the temporal dynamics, domestic Zika case percentages in 50 states and DC were 10.8% (560/5168), 26.0% (1343/5168), 52.8% (2728/5168), and 10.4% (535/5168) in the 4 quarters, and case percentages in 50 states, DC, and overseas territories were 1.70% (662/38,885), 5.91% (2298/38,885), 58.46% (22,732/38,885), and 33.92% (13,189/38,885) in the 4 quarters (Figure 3). Data were obtained from the CDC Morbidity and Mortality Weekly Report [19]. Thus, the Zika epidemic dynamics was substantially different from the CDC’s tweeting dynamics in 2016, as Zika case counts were actually the lowest in the first quarter of 2016.

**Figure 5:**
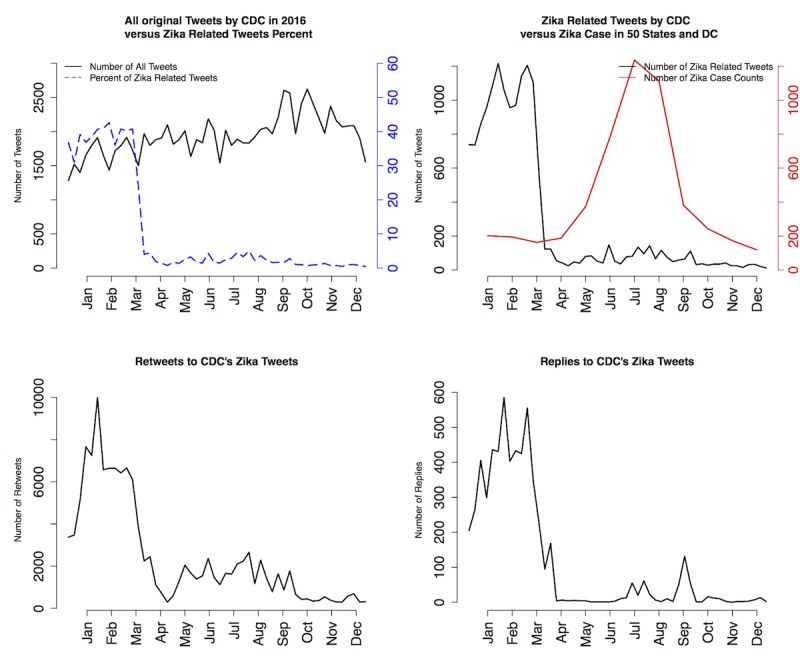
Noncongenital Zika virus disease cases in 50 states/DC and both 50 states/DC and territories in 2016. CDC: Centers for Disease Control and Prevention.

Zika was unequivocally the most tweeted health topic of the CDC in the first quarter and was mentioned in almost 50.0% (3052/6104) of all tweets in that quarter, dwarfing both HIV/AIDS- and sexually transmitted disease-related tweets; this substantial temporal heterogeneity was also demonstrated by the distinct ARIMA models in each quarter (see Table 1, the first column for original tweets). The optimal ARIMA model in the first quarter was with parameter *p, d, q*=2, 0, 3, indicating that the optimal time series model with the minimized AIC value for original tweets did not need differencing (*d*=0, order of differencing being 0, that is, already stationary and does not need further differencing), and with autoregressive and moving average term *p*=2 (indicating autoregressive time lag of 2) and *q*=3 (indicating moving average order of 3), respectively. The parameters associated with optimal ARIMA models in the next 3 quarters were *p, d, q*=2, 1, 3 (second quarter), 1, 1, 1 (third quarter), and 2, 0, 3 (fourth quarter), respectively.

Retweets of and replies to the original Zika tweets from the CDC generally followed the similar temporal characteristics, where the first quarter had the largest number of both retweets and replies (Figure 2, lower left and lower right, respectively). The optimal ARIMA models were again distinct across the 4 quarters in 2016, for both retweets (Table 1, the second column) and replies (Table 1, the third column). The only similarity was retweets in the first and the second quarter, both of which had the same parameterization (*p, d, q*=2, 1, 3). Comparing among ARIMA models for original tweets, retweets, and replies, there were only 2 pairs with the same model parameterization—original and retweets in the second quarter (both with *p, d, q*=2, 1, 3) and retweets and replies in the third quarter (both with parameter values *p, d, q*=2, 1, 2). These results revealed a substantial temporal variability across different quarters of 2016 and among original tweets, retweets, and replies.

### Multivariate Time Series Analysis Results

**Figure 4:**
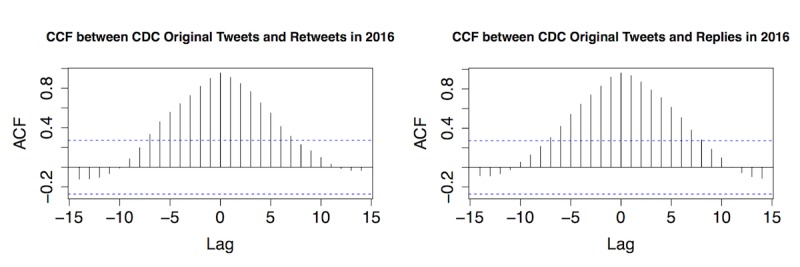
The cross-correlation function (CCF) between original Centers for Disease Control and Prevention (CDC) Zika tweets, retweets, and replies in 4 quarters of 2016. ACF: autocorrelation function.

As shown in Figure 4, strong temporal correlations were discovered between original Zika tweets from the CDC and retweets, as well as between original Zika tweets from the CDC and replies in all quarters of 2016. Most retweets and replies were centered at zero, indicating that general public’s interaction with original CDC tweets was usually synchronized. Figures 5-7 provide the plots of the CCF between Zika case and each of the following variables: original Zika tweets from the CDC, retweets, and replies in each quarter of 2016, respectively.

**Table 1:** Mutual Shannon information entropy, Autoregressive Integrated Moving Average or Autoregressive Integrated Moving Average with External Variable model parameters, and Akaike Information Criteria values in different quarters of 2016.

Quarters		Original + Case	Retweeting without commenting + Case	Reply + Case
**Q1**				
	Mutual Info	0.04	0.01	0.09
	ARIMA(X)^a^ Par	2, 0, 3	2, 1, 3	2, 0, 2
	dAIC^b^	–2.25^c^ (976.61, 974.36)	–1.88^c^ (1341.51, 1339.63)	–1.21^c^ (950.05, 948.84)
**Q2**				
	Mutual Info	0.13	0.17	0.29
	ARIMA(X) Par	2, 1, 3	2, 1, 3	0, 1, 1
	dAIC	0.96 (722.54, 723.50)	–0.88^c^ (1207.14, 1206.26)	1.88 (709.18, 711.06)
**Q3**				
	Mutual Info	0.02	0.08	0.02
	ARIMA(X) Par	1, 1, 1	2, 1, 2	2, 1, 2
	dAIC	1.95 (719.51, 721.46)	1.82 (1172.01, 1173,83)	–0.62^c^ (738.76, 738.14)
**Q4**				
	Mutual Info	0.01	0.07	0.01
	ARIMA(X) Par	2, 0, 3	0, 1, 2	0, 0, 1
	dAIC	–0.59^c^ (453.28, 452.69)	1.62 (917.84, 919.46)	1.97 (353.23, 355.20)

^a^ARIMA(X): Autoregressive Integrated Moving Average (with External Variable).

^b^dAIC: difference in Akaike information criterion.

^c^Negative dAIC value indicates better performance of the ARIMAX model compared with its corresponding ARIMA model; hence, including Zika case counts improves the model performance.

For original Zika tweets and Zika case counts, strong temporal correlations were observed in the first, second, and fourth quarter. In the first quarter, CDC’s tweets regarding Zika preceded actual case counts for approximately 7-10 days, indicated by the substantial lag of 7, 8, 9, and 10 (Figure 5, top left). In the second quarter, CDC’s tweets were ahead of the case for approximately 2 weeks (Figure 5, top right). In the fourth quarter, CDC’s tweets were behind Zika case for approximately 1-3 days (Figure 5, bottom right). In the third quarter, there was no substantial correlation between the 2 time series. These results revealed that the CDC was very active during the early stage of the Zika epidemic (especially February 2016) on social media when the actual case number was low (Figure 2, top right).

The similar pattern was also observed between retweets and Zika cases (Figure 6). The first quarter demonstrated a strong temporal correlation between the 2, whereas there was no substantial correlation in the fourth quarter. In other words, the general public was more engaged in retweeting to help disseminate the information during the first half of 2016.

The correlation between replies and Zika cases was also explored and demonstrated (Figure 7). Replies preceded case counts for about a week in the first quarter, indicating the general public’s strong interests in discussing Zika and interacting with the CDC on Twitter; this active engagement decreased as time went by. By the fourth quarter of 2016, replies were about 10 days behind actual cases.

In addition, we calculated the mutual information to explore mutual dependence between Zika cases and each of these activities on Twitter—original Zika tweets from the CDC, retweets, and replies, from an information perspective (Table 1). In the first quarter, replies had the highest mutual information (0.09) with Zika cases, which was even higher than original Zika tweets from the CDC (0.04) and retweets (0.01). Nevertheless, all these mutual information (ie, Shannon information entropy) were low, indicating a potential discrepancy between the discussion of Zika on Twitter and actual epidemic. In the second quarter, replies, retweets, and original Zika tweets from the CDC had 0.29, 0.17, and 0.13 mutual information with Zika cases, respectively, serving as the highest mutual information of all 4 quarters in 2016. In the third quarter, retweets had the highest mutual information with Zika cases (0.08), followed by both original tweets and replies tied at 0.02. In the fourth quarter, retweets got the highest mutual information again (0.07), followed by original tweets and replies with very low mutual information (0.01). In general, retweets and replies had even more mutual information with Zika cases compared with CDC’s original Zika tweets. Thus, the CDC’s tweeting pattern was an inferior indicator of the Zika epidemic than public engagement in its tweets as illustrated by the patterns of retweets and replies.

The mutual information does not consider potential temporal characteristics such as lag or trend. Therefore, we further quantified whether including an external variable of Zika case counts could increase the ARIMA model performance (Table 1). The analysis results showed that in the first quarter, all ARIMAX models outperformed their ARIMA counterparts by a large margin (difference of AIC [dAIC]=–2.25, –1.88, and –1.21 for original Zika tweets, retweets, and replies, respectively; dAIC was the difference of AIC values between ARIMAX and ARIMA models, and negative dAIC value indicated better performance of the ARIMAX model, that is, including an external variable increased the model predictability). Although Zika case counts were the lowest in the first quarter, they still highly correlated with the temporal dynamics of Web-based discussion of Zika. Including Zika case counts only improved the ARIMAX model for retweets (dAIC=–0.88) in the second quarter, for replies (dAIC=–0.62) in the third quarter, and for original Zika tweets from CDC (dAIC=–0.59) in the fourth quarter. These findings provided further evidence to confirm the large temporal variability and differences in the CDC’s response to Zika and public engagement in their responses on Twitter.

In addition, we evaluated whether Zika case could be Granger cause of original CDC tweets, retweets, and replies, or *vice versa*. The Granger causality test revealed that case count was not Granger cause for original Zika tweets from the CDC in any quarter, and *vice versa*. Thus, the correlation between CDC’s Zika tweets and actual Zika cases was not strong. Retweets, however, could serve as Granger cause of Zika cases for order from 1 to 5 (*P*=.05, .04, .02, .01, and .04, respectively) in the first quarter; this coincided with previous findings that retweets had a very high correlation with Zika cases in the first quarter (Figure 6). Similarly, replies also served as Granger cause in the first quarter for order 3, 4, and 5 (*P*=.03, .01, and <.001, respectively). Furthermore, replies served as Granger cause again in the fourth quarter for order 1 (*P*=.04). In contrast, Zika case counts in the third quarter could be Granger cause for replies with order 2 and 3 (*P*<.001 for both orders) but not *vice versa*. This was the only exception when Zika cases served as Granger cause for Twitter discussion. It is important to note that Granger causality only provided statistical evidence for potential causality and did not guarantee actual causality. For example, replies as Granger cause in the first quarter did not mean replies to CDC’s tweets “caused” Zika cases in the United States. Therefore, we should interpret that replies preceded Zika cases and had a strong association with Zika case counts at selected orders. Furthermore, the temporal heterogeneity in Granger test results showed variability across different quarters in 2016.

**Figure 5:**
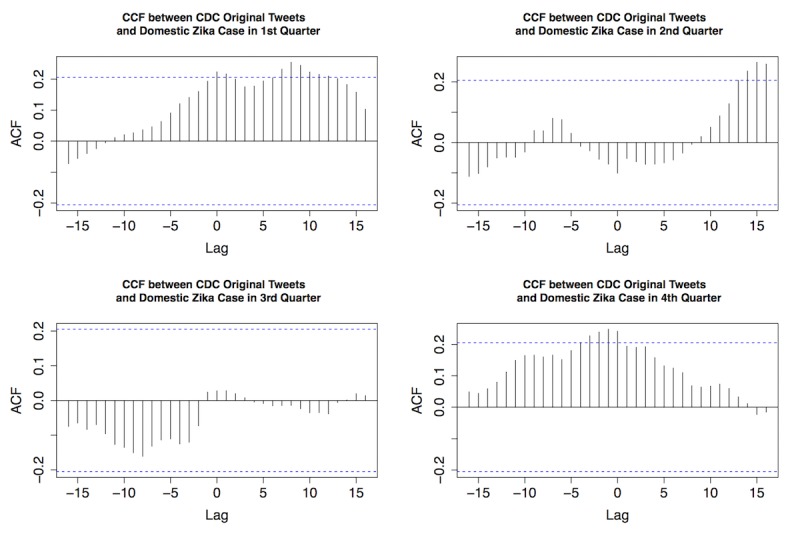
The cross-correlation function (CCF) between original Centers for Disease Control and Prevention (CDC) Zika tweets and domestic Zika cases in 4 quarters of 2016. ACF: autocorrelation function.

**Figure 6:**
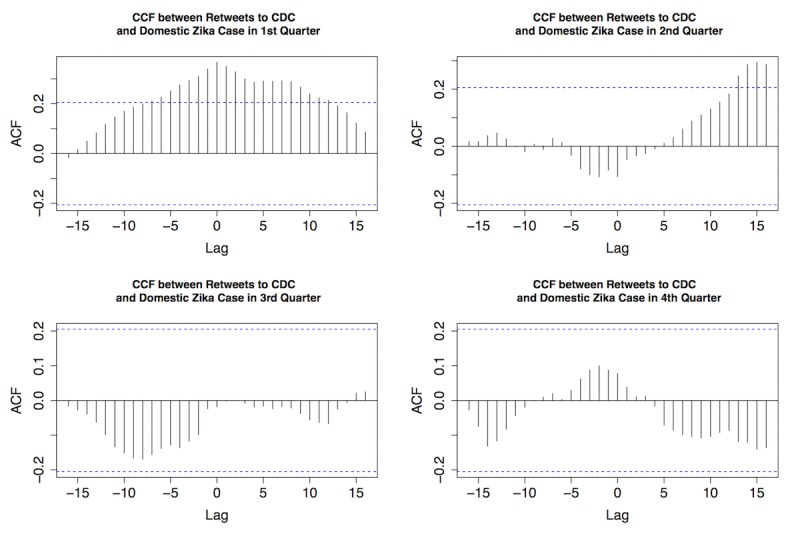
The cross-correlation function (CCF) between retweets to Centers for Disease Control and Prevention (CDC) Zika tweets and domestic Zika cases in 4 quarters of 2016. ACF: autocorrelation function.

**Figure 7:**
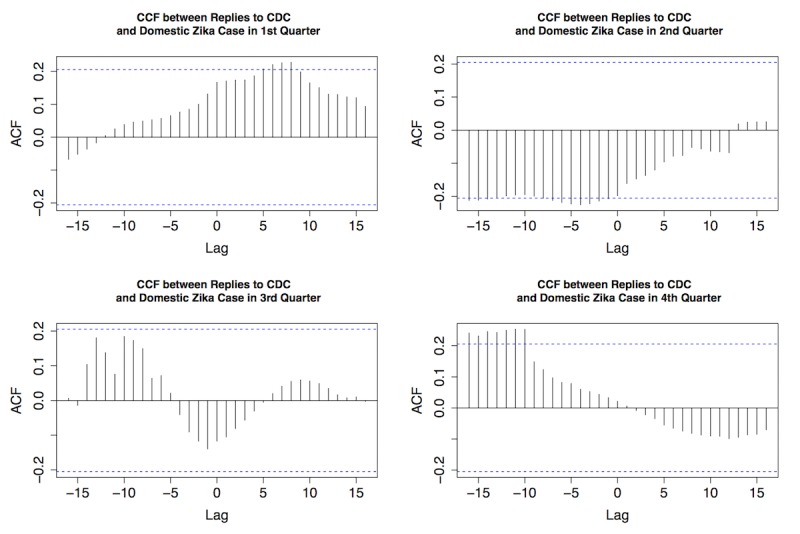
The cross-correlation function (CCF) between replies to Centers for Disease Control and Prevention (CDC) Zika tweets and domestic Zika cases in 4 quarters of 2016. ACF: autocorrelation function.

## Discussion

This study is the first of its kind that specifically investigates the temporal variability in CDC’s tweeting activities regarding Zika. More importantly, it links the temporal variability of Zika cases in the United States to that of CDC’s social media responses and public engagement in those social media messages. In general, we discovered substantial discrepancy among CDC’s tweets regarding Zika, public engagement, and actual Zika epidemic in different stages of the epidemic in 2016. As shown by our findings, there was a substantial discrepancy between CDC’s response to Zika in Twitter and the Zika epidemic. When Zika case counts were low in the United States during the first quarter of 2016, CDC was very active in disseminating information about Zika by sending out >84.0% (5130/6104) of all its 2016 Zika tweets. The CDC and its former director Dr Frieden even hosted 1-hour Twitter chat on February 16, 2016. All these activities correlated with active public engagement, as retweets and replies were also the highest among all quarters. Thus, the CDC was effective in the early warning of the upcoming epidemic of Zika and successfully gained public attention during the first quarter of 2016. However, when Zika case counts started to increase sharply in the second and third quarters of 2016, CDC’s Zika-related tweets decreased substantially and did not catch up with the Zika case counts. Nevertheless, public engagement in discussion of Zika on social media could be influenced by some other factors such as news source, personal familiarity with the disease, and potential opinion leaders who may not necessarily be health-related. All these could be future directions to expand this study.

While public engagement in CDC’s Zika tweets (ie, retweets and replies) also decreased dramatically in the second and third quarters of 2016, it was significantly associated with Zika cases, as revealed by the performance of corresponding ARIMAX models (compared with the original ARIMA models). When more case counts (including both transmitted cases and travel-related cases) were reported in Florida since late July and from Summer Olympics in Brazil between August 5 and 21, 2016, retweets and replies to CDC’s Zika tweets increased again substantially, demonstrating public’s growing and recurrent awareness of this emerging health issue. The dynamic public engagement in CDC’s Zika tweets was generally different among quarters and was also substantially influenced by and usually preceded the Zika epidemic. Therefore, public engagement in CDC’s Zika tweets was generally a more prominent predictor of the actual Zika epidemic than CDC’s tweets later in the year.

Different from previous studies that have used social media discussion trend to predict and adjust the actual disease dynamics [13,16,18,30-33], this study used Zika case counts and epidemic to infer the Twitter discussion dynamics and revealed dynamic changes throughout the year; we made this decision because the majority of domestic Zika cases in the United States were travel-related and highly stochastic [19]. Therefore, they could not be accurately captured by statistical models such as ARIMA or ARIMAX. Using social media discussion to predict the actual disease dynamics is, thus, more useful for locally transmitted diseases, such as influenza, rather than travel-related diseases.

This study has several limitations. First, we did not investigate the actual content and user identities of retweets and replies. One of the future directions is to investigate the content of these messages by using topic modeling [24] and natural language processing [34]. It will be especially valuable to examine the patterns of replies to understand the public’s responses toward the original tweets. For example, it will be interesting to examine if public responses are neutral, synergistic, or antagonistic. Another potential route was to investigate retweeting or replying network, identify potential opinion leaders, and assess their roles in disseminating health-related information from legitimate sources such as the CDC and WHO.

In this study, we focused on public engagement in CDC’s tweets (ie, retweets and replies). Nevertheless, it represents a relatively small portion of public engagement in the general topic of Zika compared with all Zika-related tweets. An extension of this study could investigate the temporal dynamics of all Zika-related retweets and replies and compare them with public engagement in CDC’s Zika tweets. Similarly, the number of original Zika tweets from the CDC were relatively low especially after the first quarter in 2016, which might influence time series analysis results (and it was also the reason we chose weekly but not a daily resolution in this study). A potential remedy was to include the temporal dynamics of all Zika-related tweets as a reference in the future study and contrast that with the CDC’s tweeting pattern.

## Abbreviations

AIC: Akaike Information Criteria
ARIMA: Autoregressive Integrated Moving Average
ARIMAX: Autoregressive Integrated Moving Average with External Variable
CCF: Cross-correlation Function
CDC: Centers for Disease Control and Prevention
dAIC: difference in Akaike information criterion
WHO: World Health Organization
